# Overcoming Immune Resistance With Radiation Therapy in Prostate Cancer

**DOI:** 10.3389/fimmu.2022.859785

**Published:** 2022-04-28

**Authors:** Arthur Mulvey, Emilien Muggeo-Bertin, Dominik R. Berthold, Fernanda G. Herrera

**Affiliations:** ^1^ Department of Oncology, Medical Oncology Service, Lausanne University Hospital, Lausanne, Switzerland; ^2^ Department of Oncology, Immuno-Oncology Service, Lausanne University Hospital, Lausanne, Switzerland; ^3^ Department of Oncology, Radiation Oncology Service, Lausanne University Hospital, Lausanne, Switzerland; ^4^ Ludwig Institute for Cancer Research – Lausanne Branch, Lausanne, Switzerland

**Keywords:** prostate cancer, radiation therapy, immunotherapy, immune checkpoint inhibitors, tumor micro-environment, cold tumor, abscopal effect

## Abstract

Prostate cancer is the second most common cancer in men and represents a significant healthcare burden worldwide. Therapeutic options in the metastatic castration-resistant setting remain limited, despite advances in androgen deprivation therapy, precision medicine and targeted therapies. In this review, we summarize the role of immunotherapy in prostate cancer and offer perspectives on opportunities for future development, based on current knowledge of the immunosuppressive tumor microenvironment. Furthermore, we discuss the potential for synergistic therapeutic strategies with modern radiotherapy, through modulation of the tumor microenvironment. Emerging clinical and pre-clinical data suggest that radiation can convert immune desert tumors into an inflamed immunological hub, potentially sensitive to immunotherapy.

## Introduction

Prostate cancer (PCa) is the world’s second most common cancer in men and the fifth leading cause of cancer death (1). Age is the major risk factor, with a median age of 67 years at diagnosis, and few before the age of 50 (2). The majority of cases are diagnosed at the localized stage, with approximately 20% presenting with *de-novo* metastatic disease (2). The introduction of prostate specific antigen (PSA) screening has resulted in increased numbers of early diagnoses in asymptomatic men, with incidence increasing by 40% globally since 2006 (3).

Even though radical prostatectomy, external beam radiotherapy (EBRT), and brachytherapy are the definitive treatments for localized disease, relapse is common (4), particularly in high risk disease [clinical T stage of at least cT2c, Gleason score of at least 8, or PSA greater than 20 ng/ml (5)].

Much progress has been made for the treatment of patients with metastatic PCa: chemotherapy with taxanes, next generation androgen inhibition, poly (ADP-ribose) polymerase (PARP) inhibition and nuclear isotope-based treatments have all shown to have significant impact on disease outcome. However, resistance to treatment is inevitable and in the majority of cases the diagnosis of metastatic PCa will be the cause of death for the patient (6).

Immunotherapy has curative potential, and immune checkpoint inhibitors (ICI) have become central to the treatment of several cancers (7–9). However, only a small number of PCa patients have responded favourably to ICI, indicating that more research is needed (10, 11). Radiation therapy (RT) has shown to cause, in select cases, an abscopal effect, in which radiation to a metastatic deposit causes tumor regression outside the irradiated field. Radiotherapy-induced systemic anti-tumor activity appears to be immunologically mediated (12, 13), and it has been demonstrated using high-dose hypofractionated RT schemas (>5 Gray (Gy) per fraction). Nonetheless, confirmation of abscopal effects when RT is administered alone has been extremely rare in clinical practice; however, the idea of administering RT in concert with ICI has sparked expectations that the abscopal effect may be frequently achieved. Unfortunately, randomized clinical trials in PCa combining hypofractionated RT with ICI have not translated in increased survival benefits (14) renewing the interest for continuing research in this field.

As a result of advancements in radiation technology, such as stereotactic body radiation therapy (SBRT), large radiation doses per fraction (>5 Gy) can be safely administered in clinical practice and high-dose hypofractionated schemes are now considered standard-of-care (15–17). **Table 1** summarizes current standard normo-fractionated and hypofractionated-high dose radiation schemas used for primary and metastatic PCa. Hypofractionated SBRT releases immunogenic tumor associated antigens over a period of several days (18, 19), therefore ICI should be precisely timed to coincide with the peak of tumor antigen presentation following RT in order to attempt clinical translation. Similarly, the immunological effects of RT might be schedule dependent, for instance Dewan et al. showed that in mouse models 3 fractions of 8 Gy with anti-cytotoxic T lymphocyte antigen 4 (CTLA4) antibody were effective in inducing an anti-tumor immune response, able to inhibit the tumor locally and systemically, whereas 5 fractions of 6 Gy were inferior, and a single fraction of 20 Gy was ineffective when combined with anti-CTLA4 (20). Even if all of these treatment regimens are considered hypofractionated high-dose RT, it is evident that a better knowledge of the triggered immune-biology will be required before radio-immunotherapy combinations can be translated to the clinic.

**Table 1:** Radiation schemas currently used for primary and metastatic PCa.

Prostate-directed RT (15)	Number of fractions	Dose per fraction in Gy
Standard RT for primary PCa	39	2
Moderate hypofractionation for primary PCa	20	3
Ultra hypofractionation for primary PCa	5	7.25
**Metastasis-directed RT** (16, 17)		
Palliative RT	1	8
	10	3
	5	4
Ablative RT	3	18
	5	11
	8	7.5
	5	7

RT, radiation therapy; Gy, Gray; PCa, Prostate cancer.

A handful of papers have studied the use of low dose radiotherapy (LDRT): doses between 0.5-2 Gy. LDRT can potentially cause dramatic remodeling of the tumor microenvironment (TME) by upregulating cytokines and chemokines, increasing immune cell infiltration, and normalizing tumor vasculature (21, 22). These events, taken together, stimulate anti-tumor T cell responses. Recent translational research has supported this biology, with increased CD4^+^, CD8^+^ T cell, as well as T effector-memory signatures, localized to tumor islets in a PCa patient undergoing LDRT and combinatorial immunotherapy (22).

We summarize the state of immunotherapy in PCa and offer perspectives on short-term opportunities for future development of immunotherapy in PCa based on current knowledge of the immunosuppressive TME. Furthermore, we discuss how RT could improve treatment responses to immunotherapy.

### Immunotherapy in Prostate Cancer

In recent years, immunotherapy and adoptive T cell therapy has resulted in paradigm shifts in the therapeutic landscape across multiple tumors owing primarily from the development of antibodies targeting immune-checkpoints such as CTLA4, programmed death receptor 1 (PD1) and its ligand (PD-L1), and the development of commercially available chimeric antigen-receptor (CAR-T) cells in haematological malignancies (7–9, 23, 24). These remarkable results have not been replicated in the context of PCa. Except in patients with microsatellite instability, ICI has failed to demonstrate meaningful clinical benefits (25). Approximately 3% of PCa patients harbor a mutation in one of the four mismatch repair genes. This small subgroup has shown long-term responses to ICI (25).

Initial basket trials of the PD1 inhibitors, nivolumab and pembrolizumab, included metastatic castration resistant prostate cancer (mCRPC) patients, but efficacy in this tumor type was minimal (11, 26). In one of the basket trials, pembrolizumab had a response rate of 17% in selected PD-L1 positive patients (26). This enriched group accounted for only 14% of all screened patients. Keynote-199, a Phase II study compared pembrolizumab monotherapy to placebo in 258 mCRPC patients who had progressed after docetaxel. PD-L1 expression was used to stratify the cohorts. In the PD-L1 positive and PD-L1 negative cohorts, response rates were 5% and 3%, respectively (27). A recent phase III trial (IMbassador250) of atezolizumab, an inhibitor of PD-L1, in combination with enzalutamide was stopped early due to futility (28).

Single agent ipilimumab did not improve overall survival (OS) versus placebo in a phase III clinical trial of minimally symptomatic chemotherapy-naïve mCRPC patients (29). Neo-adjuvant ipilimumab stimulated an influx of T cells in prostate tumors (30). However, despite evidence of T cell recruitment and trafficking, clinical responses to ipilimumab were rare, which eludes to a profoundly immunosuppressive TME, which impedes the anti-tumor T cell response (31).

Further efforts with dual ICI resulted in the Checkmate 650 phase II trial (32), which was motivated by evidence of PD-L1 upregulation following neo-adjuvant administration of ipilimumab and androgen deprivation therapy (ADT) (33). Patients received ipilimumab 3mg/kg and nivolumab 1mg/kg every three weeks for four cycles, followed by nivolumab as a single agent until unacceptable toxicity or progression. Patients were stratified based on prior exposure to chemotherapy, with overall response rates of 25% and 10%, respectively (32). Interestingly, four patients, two in each cohort, had a complete response. Patients whose tumors expressed PD-L1 ≥1% had better outcomes (36.4% versus 12.1%). These slightly higher response rates came at the expense of increased toxicity, with higher rates of grade 3-4 adverse events (42-53%) and treatment-related deaths (4.4%).

Immune checkpoint blockade has subsequently been combined with chemotherapy. The cohort B of the Keynote-365 evaluated the efficacy of pembrolizumab in combination with docetaxel and prednisone, following progression on first-line androgen biosynthesis inhibitors (34). PSA responses were seen in 34% and the overall response rate was 23%, in those with measurable disease. Toxicity was however significant, with 44.2% developing grade 3-4 adverse effects, including five deaths due to toxicity.

Beyond PD-L1 expression and microsatellite instability, efforts are being made to identify predictive biomarkers for ICI. An exploratory analysis of the Checkmate 650 trial revealed that patients with high tumor mutational burden (TMB) and errors in DNA damage repair (DDR) had better outcomes. Patients with a TMB above or below the median (74.5 mutations/patient) had an objective response rate of 50.0% (95% CI 26.0-74.0) and 5.3% (95% CI 0.1-26.0), respectively, and an OS of 19 versus 10.1 months (32). The overall response rates in patients with DDR defects increased from 23.1% to 36.4% (32). The presence of CD8^+^ tumor-infiltrating lymphocytes (TILs) and interferon gamma (IFNγ) response signatures have been shown to be predictive biomarkers for ipilimumab response in mCRPC (35). Additional predictive biomarkers for ICI activity include lactate dehydrogenase and C reactive protein levels (36), early rise in lymphocytes and eosinophils (37), and inactivation of CDK12 (38).

Vaccine-based therapies have shown activity in the treatment of PCa. Sipuleucel-T is a first-in-class adoptive cell-based vaccine approved by the Federal Drug Administration (FDA) for metastatic PCa (39). This autologous vaccine modifies patient’s dendritic cells (DC) *ex-vivo*, resulting in the expression of a recombinant fusion protein of prostatic acid phosphatase (PAP), a prostate-cancer specific antigen, with granulocyte macrophage colony-stimulating factor (GM-CSF), an immune stimulatory protein. The engineered autologous antigen-presenting cells are then re-administered to patients, without preconditioning chemotherapy. Sipuleucel-T has been evaluated in the neoadjuvant setting before radical prostatectomy. Prostatectomy specimens contained a 3-fold increase in activated T-cells following administration of Sipuleucel-T (40). In the metastatic setting, a Phase III trial of mildly symptomatic PCa patients, without visceral metastases, demonstrated a four-month improvement in OS versus placebo, without a progression free survival (PFS) or symptomatic benefit (41). Interestingly OS was substantially longer in the African American versus Caucasian PSA-matched subgroups at 35.3 versus 25.8 months, which may probably highlight the importance of HLA polymorphisms in antigen-presentation and immune activation.

Adoptive T cell therapy is currently being explored in PCa, following the major advances seen with CAR-T cell therapy in haematological malignancies (23, 24). CAR-T cells targeting prostate-specific antigens such as PSA, prostate-specific membrane antigen (PSMA), and prostate stem cell antigen (PSCA) are currently under clinical development (42). Multiple next-generation PSMA CAR-T cells with additional immunomodulatory ligands to dampen immunosuppressive signals, such as transforming growth factor beta (TGFβ) or PD1 decoy receptors are under development (43–45). Kloss et al. have shown cellular persistence and efficacy in a PCa mouse model with their PSMA-directed CAR-T, with co-expression of a TGFβ receptor (44). Another PSMA-targeted CAR-T product, with a dominant negative TGFβ receptor, has been evaluated in a first-in-human Phase I trial of ten mCRPC patients (45). The results confirmed safety, T cell expansion, and tumor-site trafficking; a decline in PSA was seen in 6 patients (median decline -33.2%, range -11.6% to -98.3%) (45).

### An Immunosuppressive Tumor Microenvironment

Prostate cancer is an immunologically ‘cold’ tumor (32, 46). Histopathological evaluation shows an ‘immune-desert’, defined as the absence of lymphocytes, or ‘immune-excluded’ phenotype, where T cells remain trapped in the stroma without penetrating the intraepithelial tumor islets (47).

**Figure 1:**
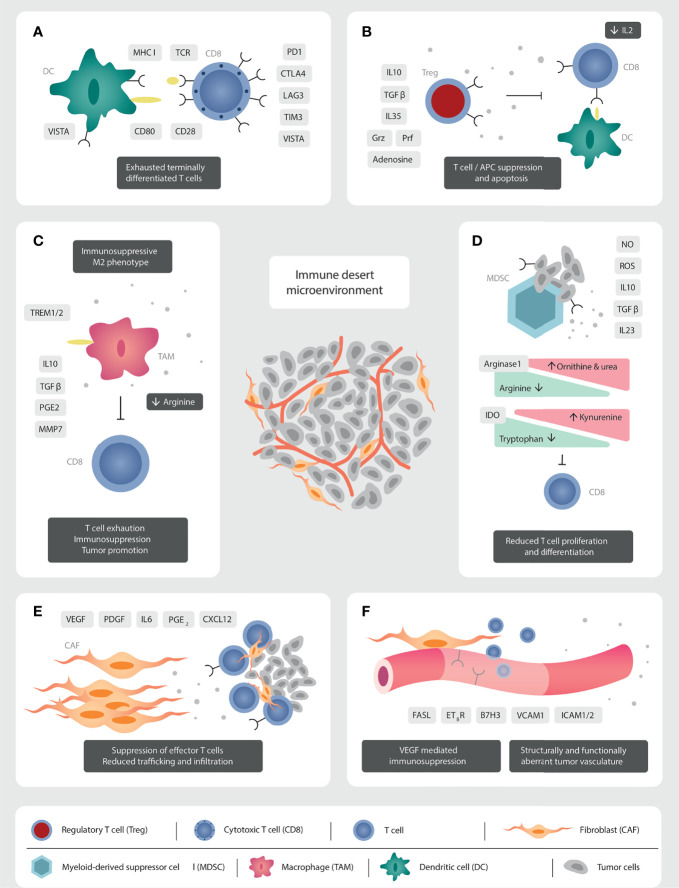
Mechanisms of immunosuppression in the prostate cancer TME. **(A)** The TILs present are functionally exhausted, expressing a terminally differentiated phenotype. High PD1/PD-L1 signalling leads to a clonally restricted TCR repertoire. **(B)** The abundant Tregs produce immunosuppressive cytokines (IL10, TGFβ, IL35), reduce IL2 concentrations through binding with CD25, and have direct cytolytic effects over T cells through granzyme and perforin secretion. **(C)** The immunosuppressive secretome of TAMs inhibit immune effector cells function and migration. Androgen receptor signalling *via* TREM plays a crucial role in production of immunosuppressive cytokines. **(D)** MDSC modulate metabolic pathways through the production of nitrate oxide and reactive oxygen species, which induce T cell anergy and apoptosis. CD8 T cell function is inhibited through the production of IL10, TGFβ, and the reduction in arginine and tryptophan concentrations. IL23 promotes androgen resistance and tumorigenesis. **(E)** CAFs reduce cell trafficking and migration through the production of a stromal extra-cellular matrix barrier. Tumor promoting CAFs inhibit immune effector cell function through the production of an immunosuppressive secretome. **(F)** Structurally and functionally aberrant tumor neo-vasculature impedes effector cells trafficking and migration. VEGF induces downregulation of vascular adhesion molecules to impede cellular anchorage and extravasation.

For those few patients with TILs infiltrating the TME, the T lymphocytes express mostly an exhausted and terminally differentiated phenotype (48, 49). In this exhausted phenotype, there is upregulation of negative checkpoints including PD1/PD-L1 (50, 51), CTLA4, lymphocyte activation gene 3 (LAG3), T cell immunoglobulin and mucin domain 3 (TIM3) and V-domain Ig suppressor of T cell activation (VISTA) (33, 48). They display low variability of the T cell receptor (TCR), due to reduced clonal differentiation of the TCR beta variable chain, which results in a restricted repertoire of peptide recognition and binding (51) **(** **Figure 1A** **).**

While the presence of intraepithelial CD8 TILs has been associated with improved survival in many solid tumors (52), this association is less defined in PCa. One study reported lower biochemical progression-free survival (bPFS) rates following radical prostatectomy in those with high intraepithelial TILs (*p*<0.37) (53). Another study of 51 men with advanced PCa with nodal metastases demonstrated a correlation between CD8^+^ TILs and poorer clinical prognosis (54). Guan et al. recently performed single cell RNA sequencing (scRNAseq) to characterize the TILs from eight mCRPC patients, progressing on enzalutamide prior to pembrolizumab treatment (55). Surprisingly, the number of CD8 T cells did not distinguish pembrolizumab responders from non-responders. Unsupervised clustering analysis demonstrated that CD8 T cell expression of inhibitory genes, and genes associated with cytotoxicity correlated with response to ICI (55), highlighting that an understanding of the functional state of TILs is crucial. In addition, this study identified a negative correlation between androgen receptor signalling and response to ICI. The scientists demonstrated that the combination of androgen receptor inhibition with pembrolizumab induced T cell activation and increased T cell polyfunctionality in their mouse model (55), supporting the potential for combinatorial ADT and ICI. However, this therapeutic approach has been evaluated in metastatic PCa patients in the IMbassador250 trial, of enzalutamide and atezolizumab, which was not shown to be clinically efficacious (28). The translational research performed in the IMbassador250 trial showed that the few patients with longer PFS treated with enzalutamide and atezolizumab had higher numbers of CD8^+^ T cell and genomic signatures linked to an immune reactive phenotype (IFN signalling, chemokines mediating T cell activation and recruitment [C-X-C motif ligand (CXCL) 9], and genes involving antigen presentation [antigen peptide transporter 1 (TAP1)] (28).

The TILs in PCa are unable to be reinvigorated with ICI, likely due to their exhausted and terminally-differentiated state, and due to the high immunological pressure dictated by an immunosuppressive TME which includes regulatory T cells (Tregs) (56–58), tumor-associated macrophages (TAMs), myeloid-derived suppressor cells (MDSC), and cancer-associated fibroblasts (CAFs) (31), and they deserve to be described here (**Figure 1**).

Regulatory T cells (Tregs) are potent suppressors of inflammation and anti-tumor immune responses. Tregs in the PCa TME constitutively express the interleukin 2 (IL2) receptor, CD25, the gene Forkhead box protein P3 (*Foxp3*), along with the immune checkpoints CTLA4, inducible T cell co-stimulator (ICOS), LAG3, TIM3, OX40, and 4-1BB (59) (**Figure 1B**). The high expression of CD25 sequesters IL2 from the TME. This impedes effector T cell activation, which requires IL2 as the third activation signal. In addition, Tregs exert their immunosuppressive function *via* the production of the potent immunosuppressive cytokines: interleukin 10 (IL10), TGFβ, and interleukin 35 (IL35) (59). Furthermore, Tregs can have direct cytotoxic effects on effector cells through the production of granzyme (Grz) and perforin (Prf) (59, 60). They alter the TME metabolic profile through the expression of CD39 and CD73, which increases extra-cellular adenosine concentrations and in turn inhibits effector T cell activation and proliferation (60). Their immunosuppressive activity is not limited to effector T cells. Tregs thwart the activity of DC through the downregulation of the crucial co-stimulatory molecule, B7, after interaction with CTLA4, and the loss of MHC class II after binding with LAG3 (60). Tregs also steer macrophages to a pro-tumoral M2-like phenotype (61), and potentiate MDSC proliferation (60) (**Figure 1B**).

Miller et al. demonstrated higher levels of intra-tumoral Tregs in PCa compared to normal prostatic tissue, in addition to increased peripheral blood Treg concentrations (56). The intra-tumoral Treg population plays a key role in tumorigenesis in early PCa, and several studies have identified CD4^+^CD25^+^ TIL concentration as a prognostic biomarker (62, 63). The analysis of 1778 PCa specimens obtained upon radical prostatectomy revealed up to 103 Tregs per 0.6 mm tissue, with the highest Treg numbers correlated with higher rates of PSA-recurrence (*p*=0.0151) (62). Furthermore, a higher number of intra-tumoral Treg cells was associated with a more advanced tumor stage (*p*=0.0355) and higher Ki67 labelling index (*p*<0.0001) (62).

Tumor-associated macrophages are a dynamic and versatile cellular group whose functions include phagocytosis and antigen presentation, in addition to regulating TME homeostasis (64). They exist across a polarised spectrum, with the M1-like phenotype secreting pro-inflammatory cytokines and chemokines, and the M2-like phenotype mediating immunosuppression *via* the production of IL10, TGFβ, prostaglandin E2 (PGE2) and matrix metalloproteinase-7 (MMP7) (65). Hypoxia-induced lactate, a product of tumoral anaerobic metabolism, is a key inducer of the M2-like phenotype (66). The dynamic TAM phenotype is also regulated *via* the C-X-C chemokine receptor type 2 (CXCR2) axis. Inhibition of CXCR2 induced a pro-inflammatory TAM phenotype and prevented PCa growth in Phosphastase and Tensin Homolog deleted on Chromosome 10 (PTEN)-deficient mice (67).

TAMs impair effective T cell migration and infiltration through the stromal tissue promoting the immune-excluded or desert phenotype (68). Being of myeloid origin, TAMs play a role in amino acid metabolism, along with MDSC. They express the enzymes arginase-1 and 2 that degrade arginine, necessary for T cell activation and proliferation (69) (**Figure 1C**).

The presence of TAMs is a predictive biomarker for biochemical relapse after radical prostatectomy in localised PCa (70, 71). The M2-like phenotype (CD163^+^) correlate with more aggressive tumors with higher Gleason score, higher metastases rates and lower cancer-specific survival in a 234 patient Swedish cohort (72). While the M1-like phenotype (CD204^+^) TAMs are associated with a lower T stage in 135 patients from a Japanese cohort (73).

Emerging evidence identifies the importance of the macrophage cell surface receptor Triggering Receptor Expressed on Myeloid cells (TREM) in PCa tumorigenesis. Androgen receptor signalling in TAMs produces anti-inflammatory cytokines, which is mediated by TREM1 (74). Indeed transcriptomic analysis of 491 localised PCa revealed shorter disease-free survival (DFS) in men with high TREM1 expression compared to low (*p*=0.0042) (74). TREM2 expression has also recently been shown to be inversely correlated to prognosis in multiple cancers. Targeting of TREM2 with monoclonal antibodies reduced tumor growth and increased response to anti-PD1 blockade in a pre-clinical model (75) and could offer potential for combinatorial treatments in PCa (**Figure 1C**).

Another cellular hallmark of the TME are the MDSC. This heterogeneous group of immature myeloid cells with strong immunosuppressive capacity are classified into two major groups: granulocytic or polymorphonuclear (PMN)-MDSC (CD11b^+^ CD14^-^CD15^+^ or CD11b^+^CD14^-^CD66b^+^) and monocytic MDSC (CD11b^+^CD14^+^HLA-DR^low/-^CD15^-^) (76, 77). PMN-MDSC share morphological and phenotypic characteristics of neutrophils, whereas monocytic MDSC are similar to monocytes. Comprehensive transcriptome and cell profile analyses identified MDSCs as a prominent TME population in PCa tumors with PTEN/Smad4 deficiency (78). MDSCs manipulate cellular metabolic pathways through the production of nitric oxide (NO), reactive oxygen species (ROS) and peroxynitrite (PNT), which inhibits effector T cell proliferation and induces T cell apoptosis (76). Feng et al. identified a crucial protein of the T-cell receptor signaling pathway, named lymphocyte-specific protein tyrosine kinase (LCK), which is nitrated by MDSC in a mCRPC mouse model, impairing T-cell function and mediating resistance to ICI (79). The enzyme arginase-1 metabolizes L-arginine to L-ornithine and urea. Myeloid cell arginase-mediated L-arginine depletion profoundly suppresses T cell immune responses and this has emerged as a fundamental mechanism of tumor-associated immunosuppression (77). MDSC-induced indoleamine 2,3-dioxygenase (IDO) decreases tryptophan concentrations in the TME (77). IDO converts tryptophan to kynurenine, depleting tryptophan concentrations which are required for T cell proliferation, and eventually results in T cells stagnating in G0 phase (76). IDO activity is increased in the PCa TME compared to benign inflammatory states, such as benign prostatic hyperplasia, and is a predictive biomarker for relapse after radical prostatectomy (80).

Adenosine levels are increased through the cell surface expression of CD39, which cleaves adenosine triphosphate (ATP) to adenosine monophosphate (AMP), and CD73, which in turn converts AMP to adenosine. This free extra-cellular adenosine inhibits effector T cells and favours Treg immunosuppressive activity *via* the adenosine receptors 2A and 2B (76). The major anti-inflammatory cytokines produced by MDSC are IL10 and TGFβ which are also highly immune suppressive (76). Furthermore Calcinotto et al. recently demonstrated the key role of interleukin 23 (IL23) secreting MDSCs in androgen resistance and tumor progression in castration resistant mice (81). The administration of an anti-IL23 alone or with enzalutamide was highly effective in reversing the resistance to castration and increased mice OS (81).

Myeloid-derived suppressor cells also have direct cellular inhibition of natural killer (NK) cells *via* membrane bound TGFβ (82), in addition to inhibition of DC and B cells (76). MDSC recruitment to the TME is mediated by tumor-derived pro-inflammatory signals. Toll-like receptor 9 (TLR9) expression by PCa cells, which appears central to tumorigenesis, stimulates recruitment of PMN-MDSCs *via* the proteins S100A8 and S100A9, and upregulates the transcription factor STAT3 which in turn inhibits CD8 T cell anti-tumor activity (83). This chemotactic pathway can be counterbalanced through direct inhibition of the STAT3 transcription factor (83). Chemokines responsible for MDSC recruitment include CXCL5 (78), and interleukin 8 (IL8), which also induces castration-resistance and tumorigenesis (84). Inhibition of IL8 in addition to ICI delayed castration resistance and increased CD8^+^ T cell infiltration in PCa murine models (84). Furthermore direct inhibition of MDSC *via* the multikinase inhibitor, cabozantinib, has demonstrated anti-tumor efficacy in mCRPC mouse models, when combined with anti-CTLA4 and anti-PD1 monoclonal antibodies (85) (**Figure 1D**).

The last key cellular group are CAFs. They are the most abundant cell in the peri-tumoral stroma (31). Their main function is the production of the extra-cellular matrix, which acts as a physical barrier, impeding trafficking and infiltration of immune-effector cells (86). In addition to providing structure, the stroma plays an essential role in tumor metabolism and waste removal (87). CAFs exhibit cellular plasticity, responding to TME-induced pressures, with epigenetic modulation resulting in cellular heterogeneity along a spectrum between tumor-promoting and tumor-restraining phenotypes (86). One study identified three different CAF subpopulations after transcriptomic analysis of 13 prostate tumors (88). They generate an immunosuppressive milieu through the production of cytokines, such as interleukin 6 (IL6) (89), growth factors such as vascular endothelial growth factor (VEGF) and platelet-derived growth factor (PDGF), chemokines such as CXCL12, as well as matrix metalloproteinases, TGFβ and PGE2 (86) (**Figure 1E**).

In addition, PCa tumorigenesis and castrate resistance is promoted *via* signalling through cholesterol and steroid biosynthesis pathways (90). Through contact-dependant and paracrine-mediated cross-talk with tumor cells, CAFs promote metastases through epithelial to mesenchymal transition of malignant cells and the polarisation of TAMs to the M2-like phenotype (91) (**Figure 1E**).

Angiogenesis is a fundamental process to tumor growth and the establishment of an immunosuppressive TME. Cancer neo-vasculature is structurally and functionally aberrant, which produces a hypoxic environment with low pH and high interstitial fluid pressure due to altered lymphatic drainage (87). Angiogenesis is predominantly mediated *via* the VEGF family, composed of VEGF-A, -B, -C, -D and placental growth factor, which act on the three VEGF receptors (VEGFR) 1-3 (92). Catecholeaminergic signalling pathways (93), as well as PGE2 (94) are also implicated in immune suppression. Hypoxia is the main stimulus for VEGF production by tumor cells and other TME cellular components, including CAFs, TAMs, and MDSC (95). Myeloid-derived monocytes expressing Tie-2 have recently been shown to play a crucial role because the ligand for Tie-2, so-called angiopoeitein-2 (Ang-2), is produced by angiogenic tumor vessels and is a chemoattractant for Tie-2 expressing monocytes. Hypoxia upregulates Tie-2 expression on monocytes and, together with Ang-2, downregulates monocyte anti-tumoral functions (96). VEGF mediates an immunosuppressive effect through various mechanisms. Firstly, effector T cell trafficking and infiltration into the TME is reduced due to the abnormal tumor vasculature, which reduces T cell tumor penetration. Furthermore, VEGF induces down-regulation of adhesion molecules on vascular endothelial cells, such as intercellular adhesion molecule (ICAM)1/2 and vascular cell adhesion molecule (VCAM)1 (97), needed for lymphocyte’s vascular wall migration. Secondly, binding of VEGF to its receptor (VEGFR) modulates the interconnected TME cellular members. This triggers STAT3 signalling in MDSC, recruitment of Tregs and inhibition of CD8^+^ T cells and DC (98). High stromal expression of VEGF receptor 2 in prostatectomy specimen is a predictive biomarker for relapse (99).

Finally, the vasculature of solid tumors expresses Fas ligand, which is a death receptor (Fas-L, also called CD95L). VEGF-A, IL-10, and PGE2 all work in concert to increase Fas-L expression in endothelial cells, allowing endothelial cells to kill CD8^+^ T cells while leaving Tregs unaffected (100). Paracrine tumor processes can thus create a tumor endothelial barrier to T lymphocytes (100) (**Figure 1F**).

In addition to the immunosuppressive cellular interplay of the TME, tumor intrinsic factors also contribute to the cold tumor phenotype. Because of the low frequency of somatic mutations, the number of immunogenic neo-antigens has also been described to be relatively low (101). Genomic mutations in the tumor suppressor gene PTEN, present in 49% of mCRPC patients (102), leads to activation of the phosphatidylinositol 3-kinase (PI3K)-AKT-mammalian target of rapamycin (mTOR) pathway and is strongly associated with an aggressive phenotype and adverse oncological outcomes (103). Loss of *PTEN* function impedes effector T cell trafficking, inhibits autophagy, and promotes resistance to immunotherapy (104). The therapeutic potential of this axis has been evaluated with everolimus, an mTOR inhibitor, which failed to improve survival in addition to carboplatin for patients with mCRPC in the post-docetaxel setting (105). This therapeutic approach deserves further investigation considering recent evidence of improved efficacy of checkpoint inhibitors when combined with a selective PI3Kβ inhibitor in melanoma murine models (104).

The downregulation of MHC class I on tumor cells reduces antigen presentation and facilitates cancer immune editing (106). Prostatic acid phosphatase, present in malignant prostate cells and normal prostatic epithelium (107), increases extra-cellular adenosine concentrations, which inhibits effector T cell and DC function, and stimulates T regs, CAFs and MDSCs (31).

This profoundly immunosuppressive and interconnected TME underpins the poor clinical results of ICI. These adaptations highlight the complex cellular and metabolic signalling pathways that immunotherapies need to overcome to achieve an anti-tumor immune response. Thus, innovative immunotherapy strategies are needed to obtain clinically meaningful patient outcomes.

## Re-programming a Cold TME With Combinatorial Therapies

**Figure 2:**
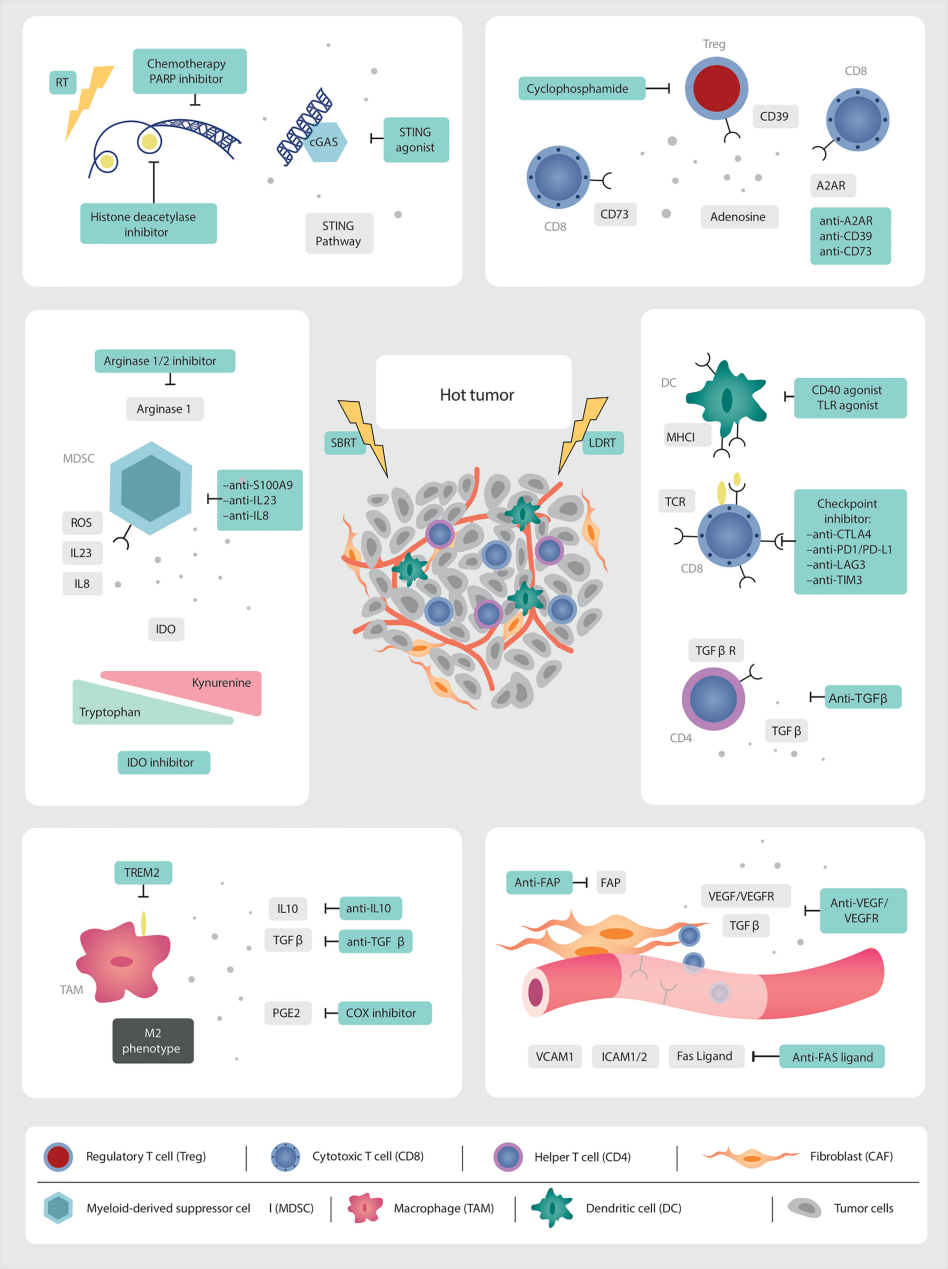
Targets for multi-modality therapeutic strategies. As we gain further granularity on the dynamic interconnected TME, new therapeutic targets are being identified which, in combination with low and high dose irradiation (SBRT), can induce immune infiltration into cold tumors. Multi-modality treatment strategies are needed to overcome the abundance of immunosuppressive factors in the prostate TME. RT is a non-invasive modality with the potential to augment anti-tumor immune responses. The *in-situ* vaccination effect of high-dose RT should be boosted by promoting antigen presentation, T cell priming and activation. LDRT can remodel the TME. This needs to be leveraged through combination strategies with immunotherapy and emerging novel drugs.

Various strategies for inflaming the cold TME have been proposed to enhance response to immunotherapies in immune-excluded and “cold” non-inflamed tumors. Methods include increasing local inflammation by inducing DNA damage through RT, chemotherapy, targeted therapies, or ablative therapies with heat or cold (radiofrequency ablation, microwave ablation or cryoablation), as reviewed in (108) (**Figure 2**).

The use of chemotherapy in combination with ICI has improved response rates and OS in many tumor types (109–111), and several studies are currently underway combining docetaxel chemotherapy and ICI in metastatic castration sensitive and resistant PCa (NCT03879122, NCT03834506, NCT04100018).

The combination of PARP inhibitors with ICI is currently being explored, after improved survival was demonstrated in mCRPC patients harbouring mutations in Breast Cancer genes (BRCA)1/2 (112). There is scientific rational that PARP inhibitors are immunogenic. They induce genomic catastrophe in homologous recombination deficient tumors. The resulting cytosolic DNA fragment debris triggers IFNγ production *via* activation of the stimulator of interferon gene (STING) pathway (113, 114). A phase I/II trial evaluated the combination of durvalumab, an anti-PD-L1 monoclonal antibody, with olaparib in seventeen mCRPC patients (115). Nine (53%) had a PSA response, with a radiological response rate seen in four (44%). Similarly, pembrolizumab and olaparib were investigated in the KEYNOTE-365 cohort A phase II trial. This trial enrolled 41 docetaxel-pretreated mCRPC patients to receive pembrolizumab 200 mg i.v. every three weeks and Olaparib 400 or 300 mg capsules twice a day (116). Confirmed PSA response rate was 9% in 82 patients with a baseline PSA assessment. Median time to PSA progression was 3.7 months (95% CI, 2.8-4.4) (116). Due to the activity observed with the combination, subsequent phase III trials were designed and are currently ongoing: pembrolizumab and olaparib versus a second androgen biosynthesis inhibitor (KEYLYNK-010 NCT03834519), and nivolumab with rucaparib, docetaxel or enzalutamide (NCT03338790).

Innovative strategies with new immune stimulatory agents are also being evaluated, such as STING-agonists in combination with ICI (NCT03956680, NCT03843359), and Toll-like receptors (TLR) agonists (TLR3: NCT02643303).

Modulation of the tumor neo-vasculature is another potential therapeutic avenue. Inhibition of VEGF, and cell adhesion molecules such as ICAM1, increases endothelial translocation and trafficking of effector T cells into the excluded or deserted TME (108). Therapeutic successes have been seen with the combinatorial approach of anti-angiogenesis agents and ICI in other tumor types (117–119), which awaits to be reproduced in PCa (120).

Additional innovative strategies under investigation include targeting metabolic pathways in MDSC, M2-like TAMs, and Tregs, as well as augmenting the presence and function of tumor-specific effector lymphocytes (108).

The first attempts to target MDSCs were made with a drug called tasquinimod. This small drug molecule inhibits the protein S100A9, which plays a key role in the recruitment of PMN-MDSC (83, 121). In a phase II clinical trial 201 mCRPC patients were randomized to tasquinimod or placebo, allowing for crossover from the placebo arm (122). A prolongation of both the PFS and OS was seen in patients receiving tasquinimod, with hazard ratios of 0.52 (95% CI 0.35-0.78, *p*=0.001) and 0.64 (95% CI 0.42-0.97, *p*=0.034), respectively (122). A subsequent phase III trial of chemotherapy-naïve mCRPC patients randomized patients to tasquinimod or placebo, with evidence of improved PFS (HR 0.64, 95% CI 0.54-0.75, *p*<0.001) but not OS (HR 1.10, 95% CI 0.94-1.28, *p*=0.25) (123). Despite these discouraging results, trials should now focus in combining tasquinimod with ICI to deplete MDSCs immunosuppressive activity while boosting T cell killing capacity.

Based on important pre-clinical research discussed earlier, current clinical trials are targeting MDSC in PCa. A Phase I/II of an anti-IL23 antibody in combination with enzalutamide is currently recruiting for mCRPC patients (NCT04458311). Another Phase Ib/II trial is evaluating an anti-IL8 inhibitor in combination with nivolumab and degarelix (NCT03689699). IDO is being targeted in a combinatorial strategy in a Phase I/II trial of the IDO inhibitor, epacadostat, in association with a bi-functional fusion protein against PDL1 and VEGF, an IL15 agonist and a vaccine targeting the transcription factor brachyury (NCT03493945) (**Table 2**). Similarly, therapeutic approaches to repolarize immunosuppressive TAMs are underway. A phase I/II trial of AZD5069, a CXCR2 antagonist, in combination with enzalutamide is currently recruiting patients with mCRPC (NCT03177187).

**Table 2:** MDSC-directed immunotherapy.

Method of action	Target	Agent	Ongoing clinical trials (NCT)	Phase
Depletion of MDSCs	S100A9	Tasquinimod (123)		
	Tyrosine Kinase inhibitor	Cabozantinib (85)		
Impairment of MDSC function/recruitment	IL23 inhibitor	Tildrakizumab	NCT04458311	I/II
	IL8 inhibitor	BMS-986253	NCT03689699	Ib/II
	IDO inhibitor	Epacadostat	NCT03493945	I/II

## High Dose Irradiation Triggering *in-situ* Vaccination

There is emerging evidence that RT can be an effective non-invasive approach to stimulate immunomodulatory effects at local and potentially systemic levels. Radiotherapy has been shown to initiate a pro-inflammatory cascade, correct aberrant angiogenesis, and potentially augment systemic responses to immunotherapy (124). Anecdotal reports of the abscopal effect, where the irradiation of a metastatic lesion results in the regression of lesions outside of the irradiated field, demonstrates the potential of a RT-induced systemic anti-tumor response (125). Case reports of the abscopal effect in patients resistant to ICI who responded systemically after receiving palliative RT treatment have boosted enthusiasm for using RT as an immunogenic trigger (125–127).

Pre-clinical mice models, in addition to translational clinical research, supports the hypothesis that the abscopal effect is an immunologically mediated phenomenon (13, 128). The abscopal effect was seen in mice models after administering a single fraction of RT (6 Gy), which was not reproduced in athymic mice (12). Formenti et al. reported a Phase I trial of 39 patients with metastatic non-small cell lung cancer treated with ipilimumab and high-dose RT (5 X 6 Gy or 3 X 9 Gy) to a single metastatic lesion (128). Objective responses were observed in 18% of enrolled patients, and 31% had disease control. The production of type I IFN following RT correlated with clinical responses (128). Functional analysis in one responding patient showed the rapid *in vivo* expansion of CD8 T cells recognizing a neoantigen encoded in a gene upregulated by radiation, supporting the hypothesis that one explanation for the abscopal response is radiation-induced tumor neo-antigens.

Unfortunately, randomized clinical trials have now conclusively established that abscopal effects are intrinsically incidental, even when RT is combined with ICI (129, 130).

High dose RT induces cell death due to the accumulation of double-strand DNA breaks, leading to critical DNA injury. The resulting cell necrosis and apoptosis induce the release of tumor-associated antigens, which act as damage-associated molecular patterns (DAMPs) (131). These cytosolic DNA fragments trigger activation of the STING pathway, resulting in the production of IFNγ. However, this cellular pathway can be inhibited by administering RT doses of above 12-18 Gy, through the production of DNA exonuclease Trex1, which degrades cytosolic DNA (132).

This process of pro-inflammatory cytokine production and immune cell activation is called immunogenic cell death (133–135). Necrotic cell death also produces other DAMPs which include high-mobility group box-1 (HMGB1), which activates TLR4 on antigen presenting cells (136). Cellular stress leads to membrane translocation of calreticulin from the endoplasmic reticulum, which elicits activation of DCs, who in turn secrete pro-inflammatory cytokines, such as IL6 and tumor necrosis factor alpha (TNFα) (137).

In addition, RT can exert pro-inflammatory responses in surviving tumor cells through multiple mechanisms. High dose RT stimulates the production by the tumor of pro-inflammatory cytokines, such as TNFα, interleukin 1 (IL1), IL6 and interleukin 8 (IL8) (138), increasing the recruitment of activated T cells and myeloid cells, and stimulates the maturation and recruitment of DCs (13). Antigen presentation is further facilitated through the upregulation of MHC class I molecules on irradiated cells and antigen presenting cells (18). The trafficking and infiltration of effector cells is augmented through the production of chemotactic chemokines, such as CXCL9, CXCL10, and CXCL16, and the upregulation of vascular adhesion molecules ICAM and VCAM (139).

## Combination of High Dose Irradiation With Immunotherapy

While single-agent ipilimumab showed no survival benefit versus placebo for mCRPC chemotherapy-naïve patients (29), another phase III trial combined ipilimumab with a single fraction of RT (8 Gy) in the post-chemotherapy setting (14) and showed a non-statistically significant improvement in OS at a median follow-up of 11 months (14). This trial randomised patients, who have progressed after docetaxel, to receive ipilimumab 10 mg/kg or placebo every three weeks for up to four cycles, following the administration of a single fraction of bone-directed RT (8 Gy) (14). Final analysis results reveal however a crossing of the curves at 7-8 months, and subsequent sustained survival in the ipilimumab and RT arm, with 4-year OS of 10% versus 3.3% (140). The high dose RT administered prior to ICI may have played a synergistic role in the delayed separation of the curves seen in this study (14).

Analysis of the combination of sipuleucel-T and ipilimumab in mCRPC patients revealed improved radiographic PFS in those with prior high dose external beam RT or brachytherapy to the prostate (141). In addition, translational analysis showed higher PD1 and VISTA expression levels in circulating peripheral T cells, and lower CTLA4 expression (141). This is evidence of long-term immune modulation following high dose RT. Similarly, long-term immune modulation by high dose RT was observed in patients undergoing prostate-directed SBRT (36.25 Gy in 5 fractions) for localised disease (142). In this study patients peripheral blood mononuclear cells produced IFNγ *in vitro* upon exposure to known prostate specific antigens (PSA, PSCA, and PSMA) at day 40 post SBRT (142). This study shows that an RT-induced *in-situ* vaccination in PCa might be observed several weeks after treatment.

Considering the multitude of potent interconnected immunosuppressive pathways activated in PCa, it is unlikely that RT to a single lesion without any additional immunological intervention will induce a sustained systemic anti-tumor response, except in exceptional circumstances. The inflammatory trigger must boost systemic innate and adaptive immunity and dampen immunosuppressive signals to alter the TME composition of distant lesions, which is determined by local interactions between the host and the genetic and epigenetic states of often clonally distinct tumor deposits. Decades of cancer vaccine failures show that all distant lesions require local reprogramming of the TME in addition to eliciting a systemic immune response (143). This explains why abscopal effects are rarely observed in clinical trials, despite SBRT-induced *in-situ* vaccination being observed primarily in pre-clinical models. Irradiation of all metastatic deposits could overcome this tumor local immune resistance, however this approach is not feasible due to severe toxicity of high dose irradiation when irradiating multi-metastatic deposits.

Thus, innovative strategies must be employed to potentiate an abscopal effect. The *in-situ* vaccination response requires bolstering through combinatorial immunotherapeutic approaches to counteract the potent immunosuppressive TME. Combination of RT and immunotherapy trials that targets innate and adaptive immunity are currently underway. In the oligometastatic setting, SBRT and durvalumab (NCT03795207), SBRT and pembrolizumab, and ADT and a TLR9 agonist (NCT03007732) are currently enrolling. Two ongoing Phase II trials are investigating the combinations of sipuleucel-T vaccine with external beam RT (NCT01807065) and SBRT (NCT01818986) for patients with mCRPC. Another Phase II trial is evaluating the addition of a dendritic cell vaccine to high dose adjuvant RT in localised high-risk PCa (NCT02107430).

The combination of CAR-T therapy and high dose RT (20-35 Gy) is gaining scientific interest in lymphomas, due to its effectiveness as a bridging therapy (144), and the fact that it may improve responses to CAR-T cells (145). A recent study compared patients who received RT prior to CAR-T cell therapy to those who did not, and found that those who received RT prior to CAR-T cell therapy had a better 1-year PFS of 78%, compared to 44% in those who did not receive RT within 30 days of infusion, with no increase in toxicity (146). There is scientific rational that RT has the potential to augment CAR-T cell therapy efficacy through increasing target antigen expression. Preclinical data demonstrates that RT increases antigen release and priming, in a dose dependent fashion (147). Weiss et al. demonstrated that combining a single fraction of RT (4 Gy) with NKG2D-CAR-T cells enhanced the number of CAR-T cells that reached the tumor site, boosted interferon-secretion, improved therapeutic efficacy, and extended mouse survival in a glioblastoma model (148). There are currently multiple Phase I clinical trials underway targeting PSMA (NCT04249947, NCT04227275) and PSCA (NCT03873805, NCT02744287) CAR-T cells in mCRPC, however a combinatorial approach with concurrent RT warrants evaluation.

Radionuclide therapy in combination with immunotherapy is currently being explored in PCa. Radionuclides like Radium-223 and Lutetium-177 (^177^Lu)-PSMA-671 selectively deliver high-dose RT, and are now standard-of-care in the management of metastatic PCa (149, 150). Emerging evidence is accumulating demonstrating their efficacy in combination with ICI in PCa. Results of Radium-223 in combination with atezolizumab were discouraging, with increased toxicity without clinical benefit (151), however strategies with ^177^Lu-PSMA-671 appear promising. Recently Aggarwal et al. reported on the use of a single dose of ^177^Lu-PSMA-671 with pembrolizumab in a chemotherapy naïve mCRPC population, with PSMA-avid disease (152). The overall response rate was 44%, with four (22.2%) patients displaying durable responses (5.4-17.8 months) (152). The PRINCE trial also evaluated ^177^Lu-PSMA-671 with pembrolizumab in the mCRPC setting (153). Thirty-seven patients, some having received prior docetaxel, with PSMA-avid FDG non-avid disease, received 6 cycles of the radionuclide in addition to pembrolizumab, 73% demonstrated a PSA response, defined as at least 50% reduction in PSA, and seven of the nine patients with measurable disease (78%) had a partial response by Response Evaluation Criteria in Solid Tumors (RECIST) (153). Radium-223 in combination with niraparib was shown to be safe and tolerable in a Phase I trial (154), with a subsequent Phase II trial pending results. Similar phase I/II clinical trials of Radium-223 in combination with olaparib (NCT03317392), and ^177^Lu-PSMA-671 with olaparib are also being conducted (NCT03874884).

## Low Dose Radiotherapy to Reprogram the Tumor Microenvironment

An innovative strategy to reprogram the TME is LDRT. There is emerging evidence that LDRT may have immune-stimulatory effects (124, 155). Radiotherapy doses of up to 2 Gy can induce DNA damage and trigger activation of the STING pathway, leading to DC activation (156). Translational research has demonstrated the upregulation of TLR signalling molecules on human monocytes after irradiation with 0.05 and 0.1 Gy (157). LDRT can remodel the TME, attenuating pro-tumorigenic TAMs, increasing effector T cell trafficking and function (124) and reducing TGFβ production (158). Furthermore, Tregs may be more sensitive to LDRT compared to effector T cells (159), and indeed low-dose total body irradiation reduced Tregs and prolonged OS in a melanoma model (160). Klug et al. reported on the immunomodulatory effect of a single fraction of LDRT in pancreatic cancer pre-clinical model, where 2 Gy reprogrammed TAMs to an anti-tumorigenic M1-like phenotype and corrected aberrant tumor vasculature allowing for correct T cell trafficking (21).

Recent pre-clinical evidence from our group demonstrates that LDRT can overcome immune resistance in immune desert tumors, including PCa, increasing T cell infiltration and response to ICI (22). Initially, we administered 0.5-2 Gy external beam LDRT to the peritoneal cavity of ovarian tumor-bearing mice, or localized 1 Gy external beam RT to subcutaneous Lewis lung carcinoma tumors (22). We discovered that 0.5 to 2 Gy RT can inflame cold tumors by increasing the frequency of CD8^+^, CD4^+^, CD11b^+^, CD11c^+^ cells in the TME (22). 1 Gy LDRT resulted in the greatest immune cell infiltration and the highest CD8^+^:Foxp3^+^ cell ratio (22). T-cell inflammation subsided after one week, but weekly cyclic 1 Gy administration resulted in ongoing immune cell recruitment into ovarian tumors.

In light of these findings, a combinatorial protocol was created, combining LDRT with ICI, nivolumab and ipilimumab, in addition to low-dose cyclophosphamide, to attenuate Tregs, and an CD40 agonist to stimulate antigen presentation (22). This orthogonal combination was administered once a week for three weeks. 83% of mice exhibited a tumor response (22). Deconvolution of the treatment protocol revealed that all agents were required for full benefit (22).

Using this encouraging pre-clinical data, we rationally designed a Phase I clinical trial for low TIL-infiltrated solid tumours (tumors with less than 5 CD8^+^ T cells per high power field by immuno-histochemistry), combining LDRT with metronomic cyclophosphamide, ICI targeting CTLA4 and PD1, together with aspirin to reduce PGE2-induced immune suppression. Overall disease control rate of the eight immunotherapy-naïve patients was 87.5%, which is striking for this immunologically cold tumor population (22). Responses were seen in PCa patients (22). A response was seen in all irradiated lesions in those who responded, and subsequent progression occurred outside the irradiated field, highlighting the critical role of locally delivered LDRT to modulate the TME.

Translational analysis confirms the inflammatory effect of LDRT with the immunotherapy combination. Pre and post LDRT biopsies revealed a ‘hot’ TME in responding patients, with increased total CD4^+^ and CD8^+^ TILs, as well as upregulation of genes involved in immune cell activation (22). Furthermore, tumors had a high levels of CD4^+^ T cell infiltration, which was associated with a significant increase in TCR clonal diversity in peripheral blood (22). Non-responders had upregulation of transcriptomic signatures associated with tolerogenic DCs and M2-like TAMs. These encouraging clinical results add weight to the RT-based combinatorial therapeutic approach.

Pre-clinical data also suggests that LDRT may enhance the efficacy of adoptive cellular therapies. DeSelm et al. discovered that LDRT of 2 Gy sensitized tumors to CAR-T cell therapy, in a murine model of pancreatic cancer with heterogeneous antigen expression (161). This augmented anti-tumor effect was not due to increasing target antigen expression on antigen-negative tumor cells but more to an immunomodulatory effect of LDRT (161). The authors found that sLeA-targeted CAR-T cells produce TNF-related apoptosis-inducing ligand (TRAIL) upon engaging sLeA^+^ tumor cells, and eliminated sLeA^−^ tumor cells previously exposed to systemic or local LDRT in a TRAIL-dependent manner (161).These results suggest a synergistic therapeutic effect of LDRT in combination with CAR-T cell therapy in solid tumors, which merits prospective evaluation in a Phase I/II trial.

Strategies of combining LDRT with SBRT have also been investigated, in attempts to maximise the systemic anti-tumor response (158). Barsoumian et al. have developed a strategy entitled ‘Radscopal’, in which high-dose RT is administered to the primary lesion, and LDRT delivered to metastatic lesions. This translational research demonstrated success across multiple mouse models, with increased survival, which was not replicated in the absence of the LDRT to metastatic sites, or high dose RT to all sites. Reponses were further increased with concomitant ICI, with anti-PD1 and anti-CTLA4 monoclonal antibodies. LDRT polarised TAM to the M1-like phenotype, increased NK cell infiltration and reduced TGFβ production (158). The potential of LDRT in combination with SBRT and ICI deserves exploration in future Phase I/II trials.

## Conclusion

Over the last decade, there have been significant advances in radiation and systemic therapies for localized and metastatic PCa. While these new therapies have improved survival, responses are not durable. More work is needed to render PCa responsive to immunotherapies.

As we gain granularity on the complex and dynamic TME, multiple key immune regulators have been identified. Orthogonal combinatorial therapies that target the immunosuppressive TME, while boosting innate and adaptive immunity, may achieve anti-tumor immune responses in PCa.

RT is a non-invasive modality with immune modulatory effects, which can be combined with immunotherapy for synergistic anti-tumor responses. High dose RT can trigger *in-situ* tumor vaccination. SBRT appears to be a promising approach for precisely targeting tumor deposits; however, when attempting to irradiate all metastasis in multi-metastatic patients, the high dose schemas of SBRT will pose toxicity issues. New innovative approaches include LDRT to large fields, either alone or in combination with SBRT to selected lesions (22, 158). Emerging evidence supports this alternative therapeutic method for sensitising PCa to immunotherapy (22). As our group demonstrated, the local effects of radiation on one metastasis should be expanded to all metastatic lesions in order to elicit an anti-tumor immune response (22). This approach will require integration with immunotherapy strategies, based on druggable targets upregulated by RT, that address the immunosuppressive TME, while also engaging RT-induced immune responses.

We believe that multi-modality trials that include novel radiation strategies are needed in PCa. Rapid clinical development requires robust pre-clinical tumor models, such as *in vitro* human organoids (162), and neo-adjuvant clinical trials to allow clinical and pathological evaluation of therapeutic combinations. The use of high throughput technology to interrogate tumor biopsies and peripheral blood will be critical in the search for predictive biomarkers. Identification of biomarkers which reflect the immune landscape, such as cytokines, circulating TILs, and anti-tumor autoantibodies, in addition to tumor-specific biomarkers, is essential to enrich clinical trial populations (163). Furthermore, adapting standard imaging methods to accurately capture the immune response is required to assess response to treatment. Radiomics analysis of multiple standard imaging modalities, magnetic resonance imaging (MRI), computed tomography (CT) and positron-emission tomography (PET), incorporated with machine learning and artificial intelligence, is emerging as a promising field (164).

## Author Contributions

FH devised the concept of this paper. All authors contributed to writing and revising the manuscript. AM and FH designed the figures. All authors contributed to the article and approved the submitted version.

## Funding

Open access funding was provided by the University of Lausanne.

## Conflict of Interest

The authors declare that the research was conducted in the absence of any commercial or financial relationships that could be construed as a potential conflict of interest.

## Publisher’s Note

All claims expressed in this article are solely those of the authors and do not necessarily represent those of their affiliated organizations, or those of the publisher, the editors and the reviewers. Any product that may be evaluated in this article, or claim that may be made by its manufacturer, is not guaranteed or endorsed by the publisher.
