# Erratum: Overcoming immune resistance with radiation therapy in prostate cancer

**DOI:** 10.3389/fimmu.2023.1185185

**Published:** 2023-03-23

**Authors:** Frontiers Production Office

**Affiliations:** Frontiers Media SA, Lausanne, Switzerland

**Keywords:** prostate cancer, radiation therapy, immunotherapy, immune checkpoint inhibitors, tumor micro-environment, cold tumor, abscopal effect

An omission to the funding section of the original article was made in error. The following sentence has been added: “Open access funding was provided by the University of Lausanne”.

The original version of this article has been updated.

